# Spiers Memorial Lecture: Emerging semiconductors viewed through the lens of a scanning electron microscope

**DOI:** 10.1039/d6fd00115g

**Published:** 2026-08-27

**Authors:** Robert W. Martin

**Affiliations:** a Department of Physics, SUPA, University of Strathclyde Glasgow G4 0NG UK r.w.martin@strath.ac.uk

## Abstract

As new materials are developed, or as their applications develop, it is important to have a range of characterisation techniques to understand their properties with a view to improving their performance. This paper considers a suite of characterisation techniques based on scanning electron microscopy to study emerging semiconductors or semiconductors with emerging applications, with an emphasis on combining multiple different techniques ideally measured simultaneously. Cathodoluminescence (CL) hyperspectral imaging is used to assess the optical properties and wavelength dispersive X-ray (WDX) provides compositional information, with an electron probe microanalyser facilitating their combined use. Electron channeling contrast imaging (ECCI) and electron backscatter diffraction (EBSD) furnish structural information, and electron beam induced current (EBIC) gives electrical information. The techniques are introduced using examples from the III-nitride semiconductors, followed by examples of scanning electron beam investigations of perovskite solar absorber layers, UV-emitting aluminium gallium nitride structures and 2D materials.

## Abstract 2

The wide range of material characterisation techniques possible using a scanning electron microscope is presented and explored with a view to providing information on the optical, structural, electrical properties of emerging semiconductor materials.

## Graphical abstract

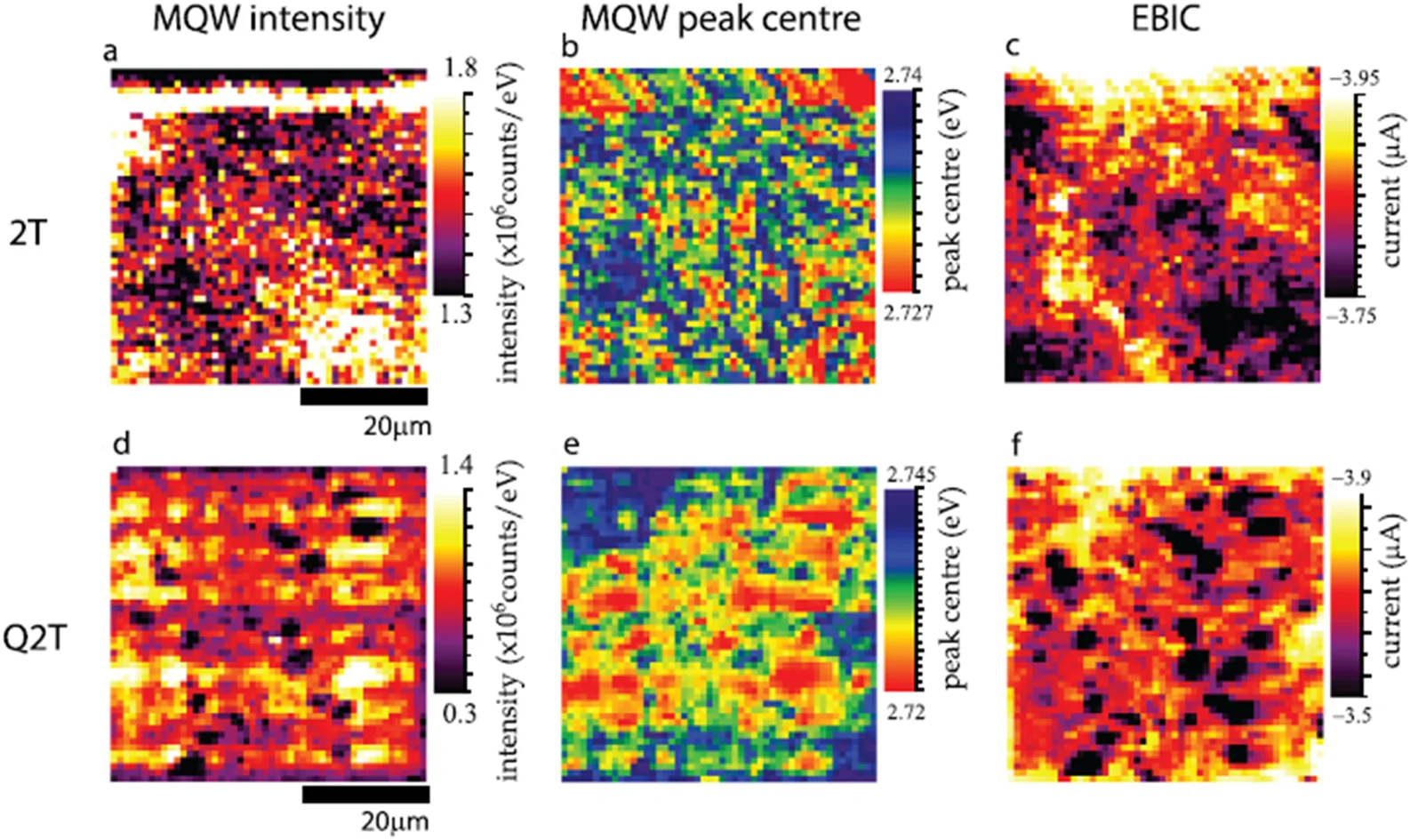

## Introduction

The number and variety of semiconducting materials have steadily increased over the years, leading to an ever-expanding range and volume of applications. Alongside new materials there are new forms of existing materials, many involving reduced dimensionality, new methods of fabrication, such as heterogeneous integration, and further reduction of defects or other deleterious features. Taken together these combine to give an expanding frontier of emerging materials as well as materials with a longer history but emerging applications. There is also an ever-expanding and advancing array of characterisation techniques for these materials, with the capability to provide information on their properties to help improve performance or functionality. Innovations and developments with characterisation techniques have led to a wide range of powerful techniques, ranging for example from transmission electron microscope (TEM) or atom probe tomography (APT) techniques that can resolve down to individual atoms to rapid electrical characterisation of millions of devices patterned across a large-area wafer. On different occasions the desired information may require high resolution or large area, breakdown of material or low damage, or data from several methods simultaneously on the same area. Theoretical calculations can be key to understanding the results and also to guiding the experimental work.

The materials in question are generally of extremely highly purity and quality, with great efforts undertaken to minimise defects that could harm their performance in devices, for example by charge trapping or adverse impacts on charge conduction. Theoretical calculations are able to address properties of specific defects, for example the formation energies of point defects and defect complexes, but when it comes to the wider scale behaviour of materials they generally focus on the ideal and pure form. The computational cost and difficulty associated with calculating the impact a low density of defects has on the functionality of an optoelectronic device is extremely high. However, a significant part of the performance of emerging optoelectronic materials is controlled by local heterogeneities rather than the ideal crystal structure alone. Important types include atomic scale point defects, structural defects (dislocations, stacking faults, grain boundaries, *etc.*), composition fluctuations, and strains as well as interfaces and surfaces. It is thus very important to use the characterisation and theoretical tools to investigate the defects and inhomogeneities that are still to be eliminated from optoelectronic materials, clarifying their formation, distribution and impact on material properties. Advances in the highest resolution characterisation tools, such as TEM and APT, and in larger-scale calculations including defects within pure materials are leading to impressive progress in this respect. Scanning electron microscope (SEM) techniques also have a powerful role here due to the wide range of length-scales that can be studied (from nanometres to centimetres) as well as the wide range of modalities (*e.g.* electron imaging and diffraction, emission of X-rays and light, *etc.*), enabling correlations to be made between the various heterogeneities and defects as well as to device performance measured *in situ*.

In addition, the defects are not always critical for device performance and there is growing interest in defect tolerant materials that function well despite imperfections.^1^ For example, consider the extremely high dislocation densities in bright InGaN/GaN LEDs or the high defect densities present in high efficiency lead halide perovskite photovoltaic cells.^1^ There are considerable savings in cost and effort to be made in device fabrication when defect reduction is not so critical. When the materials are deployed in devices for satellites or space applications the defect tolerance brings in the added dimension of extended working life and reliability. Thus, the study and understanding of defects in emerging semiconductor materials or devices has wide-ranging significance, either helping to minimise the presence of harmful defects or identifying materials that are less impacted by defects.

This paper considers the application of scanning electron microscope-based techniques to study emerging semiconductors or semiconductors with emerging applications, in particular looking at the combination of different techniques ideally measured simultaneously. As interest in the optical properties of ultra-wide bandgap semiconductors, such as Al_*x*_Ga_1−*x*_N, Ga_2_O_3_, diamond, BN, *etc.*, increased there have been clear benefits in using the focussed electron beam of an SEM (or TEM), rather than ultrashort wavelength lasers, to excite optical emission. This cathodoluminescence (CL) can be used to produce highly spatially resolved spectral/hyperspectral maps using a variety of light collection systems, both bespoke and commercial.^2,3^ Samples can be cryogenically cooled or heated and modern pulsed electron gun systems allow picosecond time resolution.^4,5^ The nature of the SEM also allows CL to be imaged from regions ranging from nanometres to millimetres in size, covering advanced nanostructures to broad area devices or full wafers. This paper will describe how CL can be used in combination with wavelength (or energy) dispersive X-ray WDX/EDX to add compositional information, or with electron channeling contrast imaging (ECCI) or electron backscatter diffraction (EBSD) for structural information, or with electron beam induced current (EBIC) for electrical information.

## SEM-based studies of a previously emerging material: InGaN

Our entry into the use of combined SEM-based characterisation techniques came from the emergence of In_*x*_Ga_1−*x*_N as a highly efficient emitter of UV-visible light in the late 1990’s.^6^ This was the active material in rapidly developing UV/blue LEDs and lasers with very high internal quantum efficiencies despite high densities of threading dislocations – so high that the current wisdom suggested viable devices should be impossible. An early requirement was to relate the InN mole fraction (*x*) to the In_*x*_Ga_1−*x*_N bandgap and thus to the emission wavelength from the quantum wells, which was complicated by an incorrect value (up until 2002) for the InN bandgap as well as intense in-built electric fields and strains arising from the lack of a native substrate. Techniques such as photoluminescence and X-ray diffraction were being used to make separate measurements of the optical emission and material composition and having a characterisation tool that could simultaneously measure the InN fraction and luminescence wavelength from the same microscopic region was very desirable. We capitalised on the reflecting optical objective integral to an electron probe analyser (EPMA) to achieve this by combining CL spectroscopy and wavelength dispersive X-ray (WDX).^7^ The energy of the dominant peak in a CL spectrum could be compared with simultaneously measured WDX data. Hyperspectral mapping was employed for the CL and, in later work, principal component analysis aided extraction of the most significant features from the 3D datasets, with the possibility of including the CL spectrum and X-ray data into a combined eigenvector.^8^

Restricting the electron beam energy to approximately 5 keV or lower allowed the study of thin layers (thicknesses down to 100 nm) with a similar lateral spatial resolution. Even at this scale it was evident that the InGaN was not of uniform composition. Other groups used TEM to explore this non-uniformity on smaller length scales, bringing in the observation that the indium segregation could in fact be induced by the high energy electron beam.^9^ Subsequently APT was used to obtain a clearer picture of the atom distributions, indicating that there was no major segregation.^10–12^ However, randomness in the alloy composition still produced significant composition fluctuations, with the random alloy distribution producing lower bandgap regions with relatively high InN and wavefunction localisation effects. In addition, spatial variations in the quantised energy levels arise from fluctuations in the widths of the quantum wells that made up the light emitters.

Along this journey we employed electron beam induced current (EBIC) measurements to study micron-scale spatial variations in InGaN/GaN quantum well LEDs alongside CL (and electroluminescence, EL) within the EPMA instrument.^13,14^ The EBIC image is formed by scanning the electron beam across a region of the device while monitoring the current flowing in an external circuit to which it is connected. The internal electric fields drive some of the generated charge carriers to the circuit, counter-acted by competing channels such as defects or grain boundaries. Collecting hyperspectral CL maps at the same time as the EBIC provides further information on the behaviour of the generated charge carriers, which undergo one of three mutually exclusive processes: radiative recombination (CL), non-radiative recombination or escape to the circuit (EBIC). Four versions of InGaN/GaN quantum wells produced by metal–organic vapour phase epitaxy (MOVPE), with different choices for the quantum well and barrier growth temperatures, were studied.^14^ For example, the “two temperature” (2T) samples had the growth temperature ramped up and down between 750 °C for the wells and 900 °C for the barriers. This should increase the quality of the latter at the risk of loss of indium from the wells. An alternative, referred to as “quasi two-temperature” (Q2T) involved starting the growth of the barriers at the same temperature as used for the wells, before ramping the temperature. This aims to give the wells a protective cap before the higher temperatures come into play. TEM study had shown that quantum wells grown without the exposed anneal step (such as Q2T) showed much more uniform thickness than those with the anneal (such as 2T).^15^

**Fig. 1:**
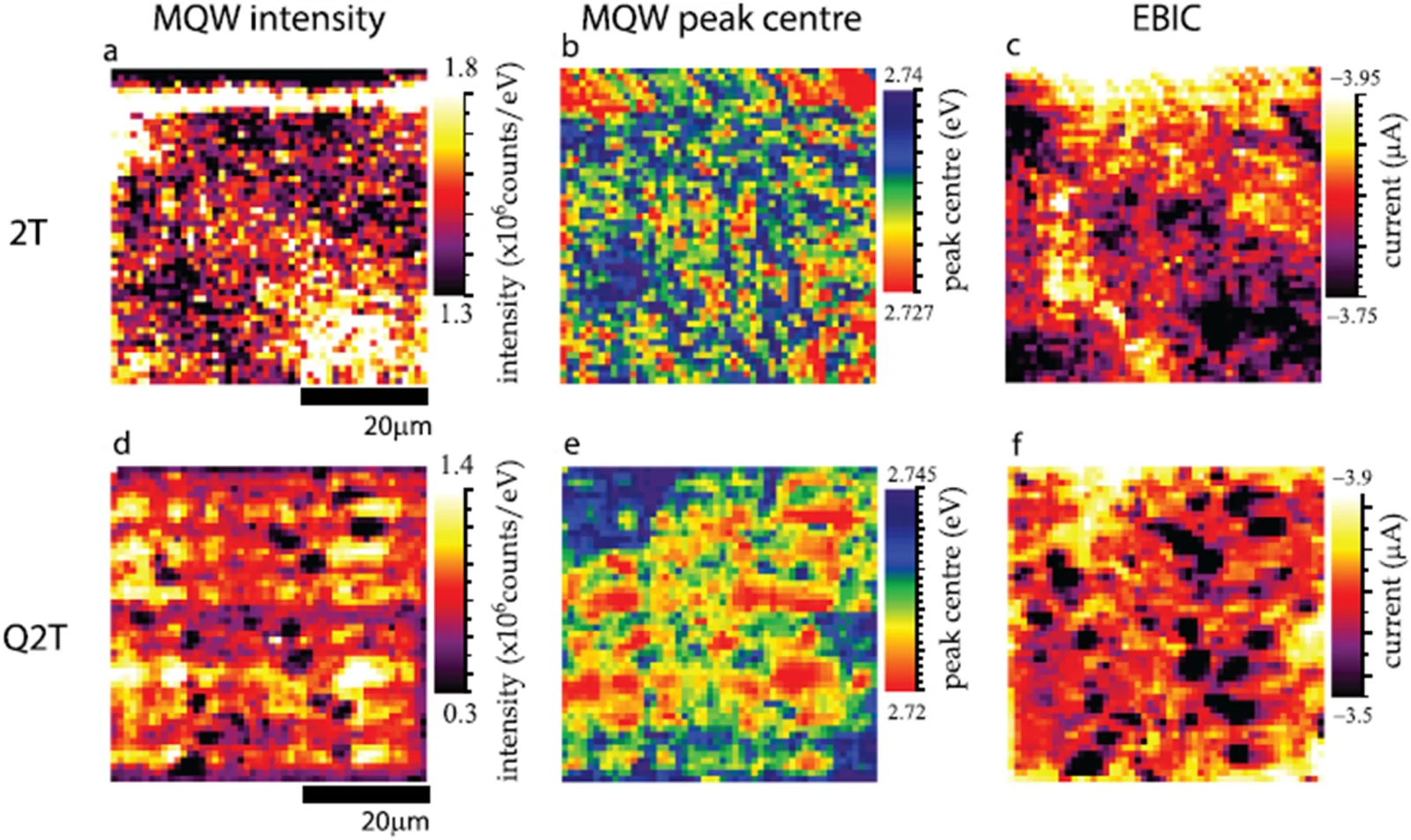
Results from a CL-EBIC study of InGaN/GaN quantum wells grown with (2T) and without (Q2T) an anneal of exposed InGaN. (a–c) Show CL intensity, CL peak position, and EBIC maps for the 2 T device and (d–f) the same for Q2T devices. Reproduced from Wallace *et al.*,^14^ licensed under CC BY.

The optical efficiency of the LEDs showed significant differences between these two cases, being noticeably higher at moderate currents for the non-uniform, annealed samples. The EBIC-CL also showed noticeable differences between the two cases. Fig. 1 compares maps of the quantum well CL intensity and peak emission energy with simultaneously collected EBIC maps from a representative sample with wells that had been annealed when exposed (2T) and those with no anneal (Q2T). The top line of Fig. 1 is for the 2T sample and shows fairly long-range (10 s of µm) variations in the CL and EBIC intensities, which are anticorrelated – compare the bright lower right region in the CL with the dark EBIC at this point. Contrast this with the Q2T results in the lower line where dark spots in the CL intensity line up with dark spots in the EBIC. This correlation is clear evidence of increased non-radiative recombination in these regions, with the injected electrons neither recombining radiatively nor reaching the external circuit. A likely cause is clusters of point defects which are annealed out in the other type of sample, albeit at the cost of non-uniform quantum wells.

Some time later CL measurements on extremely high quality InGaN/GaN quantum wells were able to spatially resolve individual point defects.^4^ This work provides an example of CL from cryogenically cooled samples, with measurements at 10 K serving to minimise carrier diffusion in the InGaN wells and increase spatial resolution of the CL maps. Two types of non-radiative defects were identified, with differing impacts of the luminescence efficiency.^4^ The authors were able to demonstrate the impact of threading locations on the optical efficiency of the quantum wells even at 10 K, with confidence that individual threading dislocations were being observed provided that the density was below 10^17^ cm^−3^.^4^

Theoretical studies often play a key role in the development of new or emerging semiconductor materials and indeed in the identification of new compounds meriting investigation. For the historical work on InGaN the theoretical studies had to contend with fluctuations in composition and quantum well widths, leading to varying degrees of carrier localisation, intense in-built electric fields, and significant strains. Beyond those there were different crystal structures and orientations and large populations of defects. Two bodies of work stand out in providing an explanation for the optical emission in visible InGaN LEDs. Schulz *et al.*^16^ used atomistic tight-binding theory to calculate the optical and electronic properties. Their analysis included fluctuations of well width, random alloy composition disorder, variations in strain and built-in fields and also Coulomb effects. They found that the hole wave functions were much more tightly localised than those of the electrons, with the latter mainly localised by well-width fluctuations whereas the hole states were already localised by random alloy disorder. David^17^ developed an optical model based on a two-band effective mass Hamiltonian, including random alloy disorder but neglecting Coulomb interactions and assuming carrier thermalisation. A large number of quantum states and configurations were calculated, with a note that earlier works may have been dependent on the number calculated and assumptions about their population. The results show an impressive match to optical spectra from blue, green and red InGaN/GaN emitters, including the broad spectral widths and current-dependent behaviour. The verdict on the localisation question, following the many experimental studies trying to shed light on this topic, was of a hybrid situation with the luminescence spectrum arising from transitions involving both localised and delocalised states.^17,18^

Having introduced the use of the scanning electron beam techniques CL, WDX/EDX and EBIC, this article will now go on to introduce further possibilities in this family, namely ECCI and EBSD, and then to describe their use in combination with other SEM-based techniques to investigate and optimise some examples of emerging semiconductor materials.

## Characterisation of semiconductors using EBSD and ECCI

### Electron channeling contrast imaging (ECCI)

**Fig. 2:**
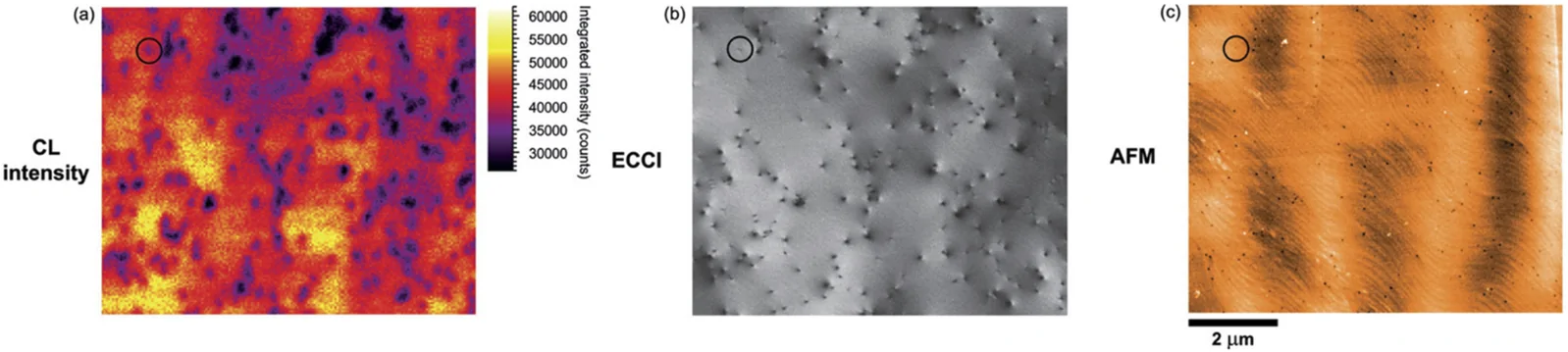
Matching sets of threading dislocations revealed by (a) CL intensity, (b) ECCI, and (c) AFM studies of the same region of a GaN sample. Reproduced from Naresh-Kumar *et al.*^19^ with permission from Oxford University Press.

ECCI is an additional SEM-based technique which highlights disruptions or modifications in the regular structure of crystalline materials to allow threading dislocations and other defects to be imaged and identified in a fast and reliable manner with minimal sample preparation. ECCI is performed by orienting the sample such that crystal planes are close to the Bragg angle with respect to the incident electron beam. The intensity of backscattered electrons is monitored as the electron beam is scanned across the region of interest and an image can be produced with contrast generated by any changes in the orientation or spacing of the selected planes, as result, for example, from the presence of a dislocation. Fig. 2 shows results from a layer of GaN, where the same set of dislocations has been imaged by ECCI and CL as well as atomic force microscopy (AFM) of a specific region of the sample.^19^ Differences in grey scale contrast in the ECC image identify regions of different tilt or twist, and threading dislocations appear as spots with black–white contrast. These exactly match the dark spots in the room temperature CL intensity map, which result from non-radiative recombination at the dislocations, enlarged by electron diffusion, and in the AFM image (taken later from exactly the same region) where the visibility of the dislocations is increased by treating the surface with silane/ammonia. The ECCI and AFM also reveal a pattern of atomic steps across the epitaxial sample, allowing some identification of the dislocation types (those terminating atomic steps being of screw or mixed type). A more complete identification of dislocation types had been demonstrated using ECCI, by repeating images with specific changes in sample orientation.^20,21^ The black-white contrast pattern for the threading dislocations was shown to change in different ways for screw, edge and mixed dislocation types between ECC images acquired from different orientations, which for GaN would be two symmetrically equivalent crystal planes whose reciprocal lattice vectors are at 120° to each other.^20^ The approach allows unambiguous identification of all the threading dislocations in the GaN film without the need to compare results with dynamical simulations of channeling contrast.

This section has introduced how ECCI can identify dislocations and provide qualitative information on tilt and twist within semiconductor layers, making it useful for identifying grain structure. However, it doesn’t go as far as quantitative information on grain orientation or strain, for which EBSD provides a powerful option.

### Electron backscatter diffraction (EBSD)

As optoelectronic devices increase in capability and performance it can become important to have as full a picture as possible of the stresses and strains within the material. EBSD is a well-established technique for structural characterisation of crystalline materials and recent advances in both analysis tools and detection systems have significantly improved its spatial resolution and sensitivity, enabling the detection of small misorientations, strain distributions and extended defects. Hiller *et al.*^22^ have recently demonstrated how EBSD can now resolve individual dislocations, identify their type and quantify relative local rotations and strain variations in a crystalline thin film. The innovations allow EBSD to achieve a misorientation resolution of ≈0.1 mrad or 0.006° and a relative strain resolution of ≈1 × 10^−4^ (or 0.1 mm m^−1^).^22^

The example material is GaN, which is the underlying material for a number of expanding applications such as high-voltage, high-temperature transistors. Dislocations propagating from such underlayers can cause leakage currents and restrict breakdown voltages. A range of characterisation techniques have been used to investigate dislocations, and resulting strains, within GaN and other semiconductor materials including TEM, X-ray diffraction, AFM, Raman and CL. EBSD analysis of strain on 3D structures in GaN was discussed in ref. 23 and 24 in combination with micro-Raman mapping, CL and ECCI. Ref. 22 goes on to show how EBSD, supported by ECCI, can give a direct measure of the strain and mis-orientations around a single threading dislocation as well as identify whether the dislocation has a screw component or is pure edge type.

The basis for EBSD is the beautiful diffraction patterns generated by backscattered electrons being diffracted as they exit a heavily tilted sample, usually at ≈70° relative to the incident electron beam. Traditionally the diffraction patterns are captured using a phosphor screen coupled to a camera, but recent developments utilise direct electron detectors with significant advantages in performance.^25^ The EBSD patterns contain multiple sets of Kikuchi bands, each of which corresponds to a particular set of crystal planes. A pattern is collected from each point in a map across the region of interest and interpreted using increasingly advanced EBSD analysis to provide detailed information on the structural properties such as strain, mis-orientation, *etc.* High quality patterns and analysis of detailed structure in them allow subtle effects to be resolved, such as differentiation of changes in crystal polarity.^26^

**Fig. 3:**
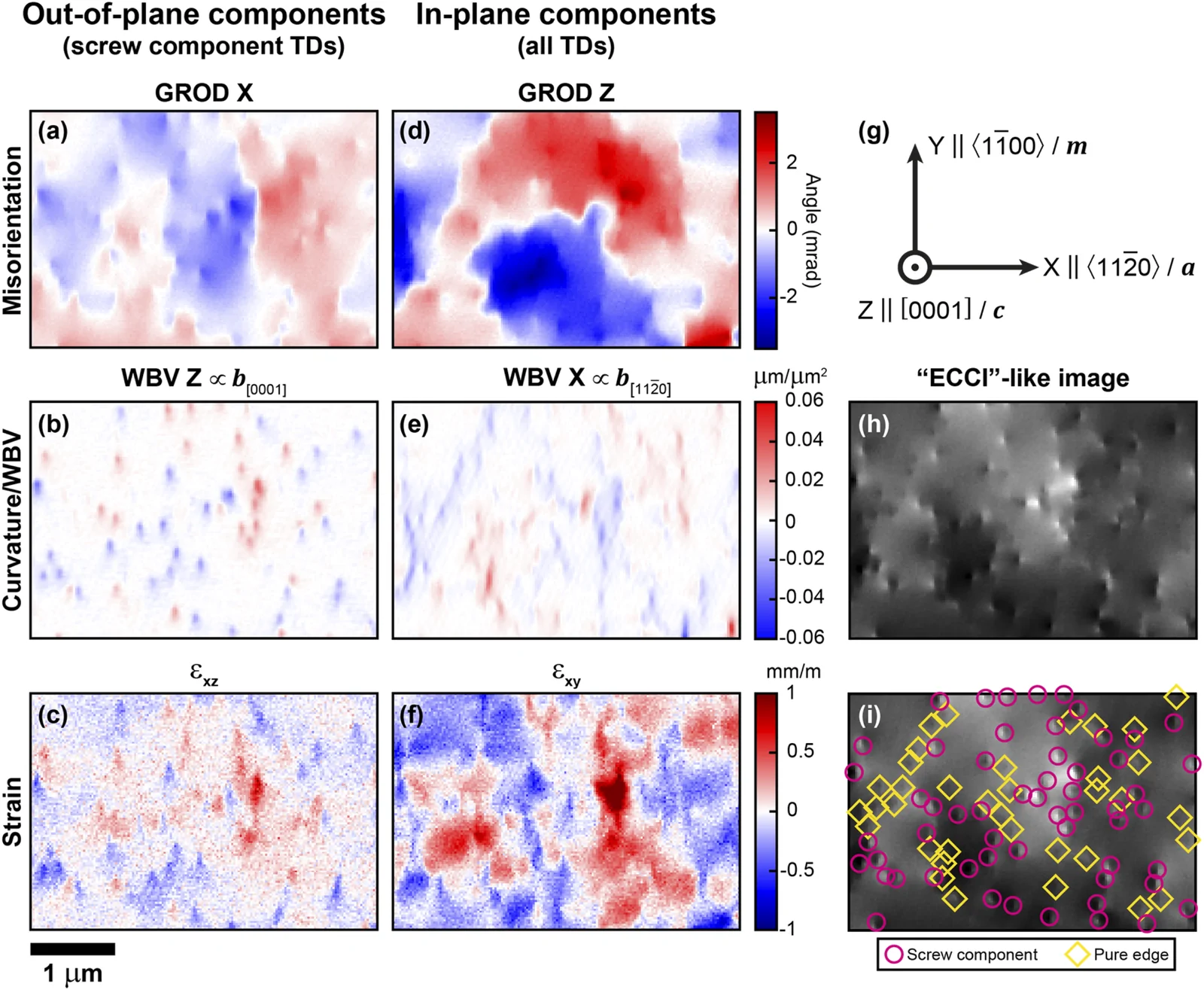
Key results from the misorientation (GROD), curvature (WBV) and deviatoric strain analysis from the EBSD measurements of threading dislocations in a GaN thin film. The left column (a–c) shows maps where screw component dislocations are resolved, whereas the right column (d–f) show maps where all dislocations are visible. (g) Shows crystallographic directions and their alignment with the sample reference frame. An ECCI-like image, constructed from the EBSD data, without and with the identified dislocations marked, is shown in (h) and (i). Adapted from Hiller *et al.*,^22^ licensed under CC BY.

The analysis in ref. 22 includes determination of the magnitude of the misorientations around a particular axis at each point in a map using grain reference orientation deviation (GROD) analysis. Plots reveal and quantify variations in misorientation and plotting its angle with respect to rotations about certain axes (in this case *Z*‖[0001] and *X*‖〈112̄0〉) allows differentiation of in-plane (GROD *Z*) and out-of-plane (GROD *X* and *Y*) rotations, as reproduced in Fig. 3. Dislocations with out-of-plane rotation were shown to require a screw component. The paper also employs a weighted Burgers vector (WBV) method to highlight this distinction, with pure screw dislocations revealed in WBV maps of the *z*-component and all dislocations evident in maps of the *x*-component. In addition, the authors calculated maps of the relative deviatoric (*i.e.* shape-changing) strain components directly from distortions of the experimental diffraction patterns when compared with dynamically simulated patterns of unstrained material. The full strain tensor is calculated with example maps of two of the shear components *ε*_*xz*_ and *ε*_*xy*_, included in Fig. 3. The EBSD is shown to arise from the top 20 nm or so of material, with a lateral resolution of ≈20 nm for the case of GaN. Therefore, the isotropic elasticity simulation of the misorientations and strain had to consider both rotations and strain in the bulk and due to surface relaxation.^22^

The results demonstrate the power of EBSD for full assessment of strains and misorientation in crystalline materials, including analysis and type identification of dislocations in semiconductor thin films.

## Characterising perovskite semiconductors using SEM-based techniques

Solar cells based on halide perovskites have demonstrated an impressive rise in photovoltaic efficiency, now reaching 28% and exceeding crystalline Si. Both inorganic and hybrid halide perovskites show non-uniformities at micron and nanometre length scales making CL an attractive tool for their study. There have been many CL studies of halide perovskites with work up to 2020 reviewed by Guthrey and Moseley.^27^ Their review and other reports highlight the issue of beam damage where reversible and irreversible change can be induced by the incident electron beam. This is particularly the case for hybrid perovskites, such as methylammonium lead iodide (MAPI) or formamidinium lead iodide (FAPI), where the beam can induce defects, elemental segregation and loss of the organic species due to localised heating. Investigations such as the work by Ferrer *et al.*^5^ have explored how the availability of CL systems with optically driven pulsed electron guns can minimise the beam damage as well as allow time-resolved CL studies of picosecond charge-carrier dynamics. They systematically studied the impact of beam conditions on triple-cation (MA/FA/Cs) double-halide perovskite films, demonstrating the benefits of the pulsed mode for countering beam damage. The time-resolved results showed shorter lived dynamics than photoluminescence of comparable films, suggesting that the stronger excitation of the pulsed electron beam is responsible for additional effects such as saturation of non-radiative channels or Auger recombination.^5^

### Characterising perovskites using CL with EBSD

**Fig. 4:**
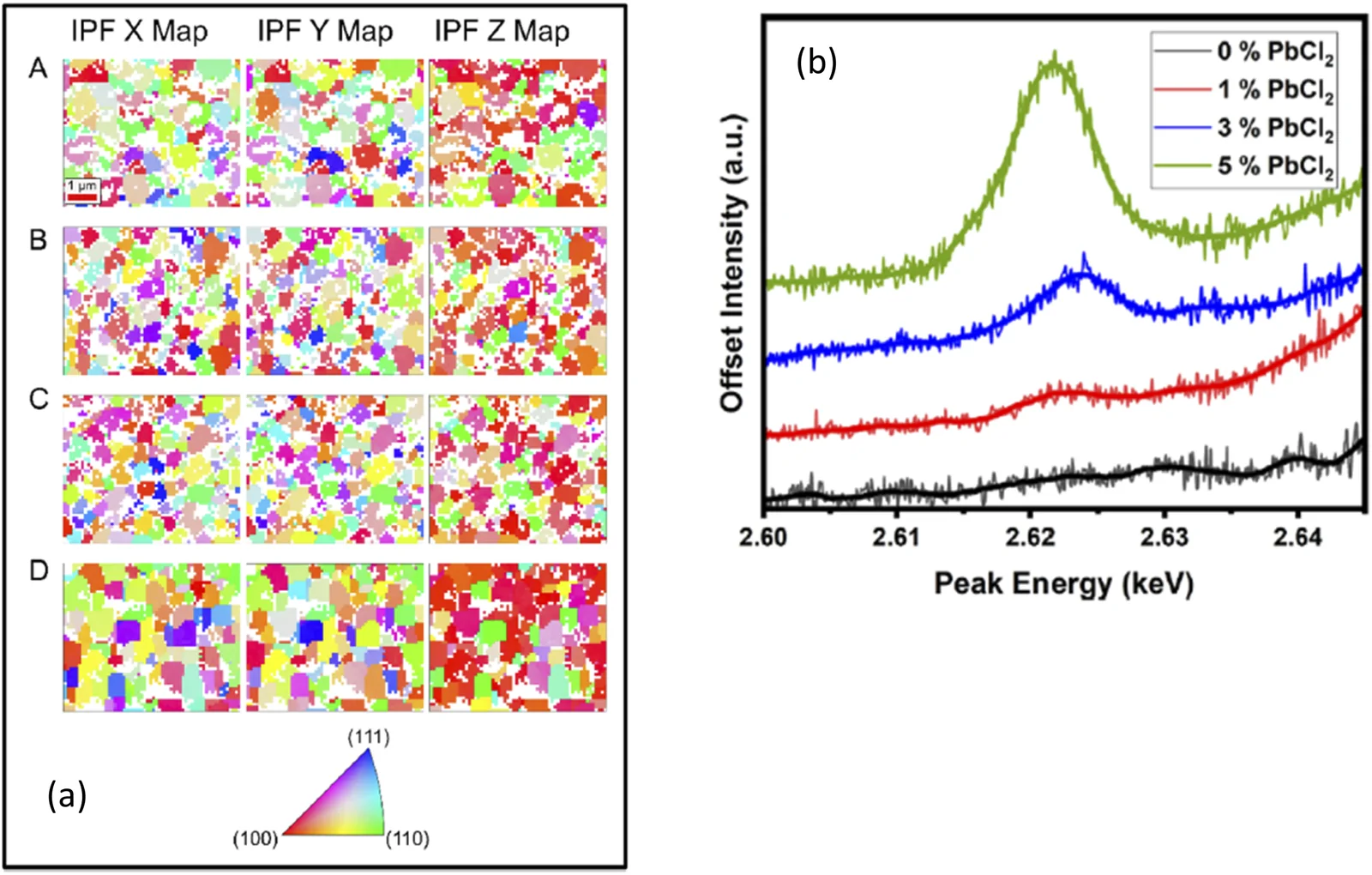
(a) Inverse pole figure (IPF) maps showing grain level crystallographic orientation for PbCl_2_ doped CsPbI2Br films – (A) 0%, 0 mg ml^−1^, (B) 1%, 1.8 mg ml^−1^, (C) 3%, 5.0 mg ml^−1^, and (D) 5%, 8.4 mg ml^−1^. (b) WDX peak for chlorine showing increasing incorporation for higher dopant content in the precursor. Reproduced from Nicholson *et al.*,^28^ licensed under CC BY.

All-inorganic perovskites, such as CsPbI_2_Br with its applications in tandem cells and indoor PV, are more robust under electron beam excitation and allow combined electron-beam investigations such as CL with EBSD or WDX. One such study looked at chlorine incorporation as this has been shown to be effective for passivating defects and/or improving stability. The grain structure and optical properties of a series of CsPbI_2_Br doped with various levels of PbCl_2_ were investigated using WDX, XRD, EBSD and CL.^28^ EBSD data revealed the impact of increased chlorine doping on grain size and crystal orientation. The inverse pole figures in Fig. 4(a) display the measured crystal directions with respect to reference directions (*XYZ* where *Z* is out-of-plane) and display the orientation of individual grains, as well as demonstrating increasing grain size with increasing Cl doping. Furthermore, the increasing dominance of red colouration in the *Z*-oriented maps show increasing preferential orientation to the [100] direction as the Cl doping increased, suggesting that the Cl dopant slows the nucleation process thereby enabling greater grain size and preferential orientation. There were conflicting reports about the actual incorporation of Cl into doped films and in this case WDX measurements were able to demonstrate a clear proportional incorporation of chloride ions, as shown in Fig. 4(b). The CL maps revealed a consistent shift of emission wavelength as would be explained by the bandgap changing due to increased chloride ion incorporation.

For chlorine-doped hybrid halide perovskites a CL study by Wang *et al.* demonstrated how crystallinity and uniformity could be optimised by using a combination of MACl and PbCl_2_ dopants.^29^ CL maps showed how the mixed dopant approach significantly reduced recombination losses at the interfaces, thereby promoting efficient extraction of charge carriers as evidenced by the very high (>26%) efficiency solar cells that were made using this approach.

In other combined CL-EBSD work Nicholson *et al.*^30^ reported correlations between optical emission and structural properties of all-inorganic CsPbI_2_Br perovskite absorber thin films prepared on a selection of electron transport layers (ETLs) processed at two different annealing temperatures. Among the different ETLs, zinc oxide was shown to promote perovskite films with enhanced grain size and preferred growth along the [100] orientation, and relatively uniform light emission for the high temperature processed layer.^30^

## Characterising ultra-wide gap semiconductors for UV applications

### UV LEDs studied using CL and EBIC

Much work has focussed on developing deep UV LEDs based on ultra wide bandgap AlGaN due to their large range of applications.^31^ Recent years have seen significant improvements in the efficiency of these devices but the state-of-art is still limited at very short wavelengths, with maximum external efficiencies near 1% for devices emitting at 235 nm or 260 nm. The large bandgaps of the required materials, which approach 6 eV, present challenges for laser-based studies of their optical properties and increase the attraction of CL and the high excitation energies available from an electron beam. The CL can be combined with EBIC, as introduced above, to provide information on the defect populations most likely responsible for the low LED efficiencies.

**Fig. 5:**
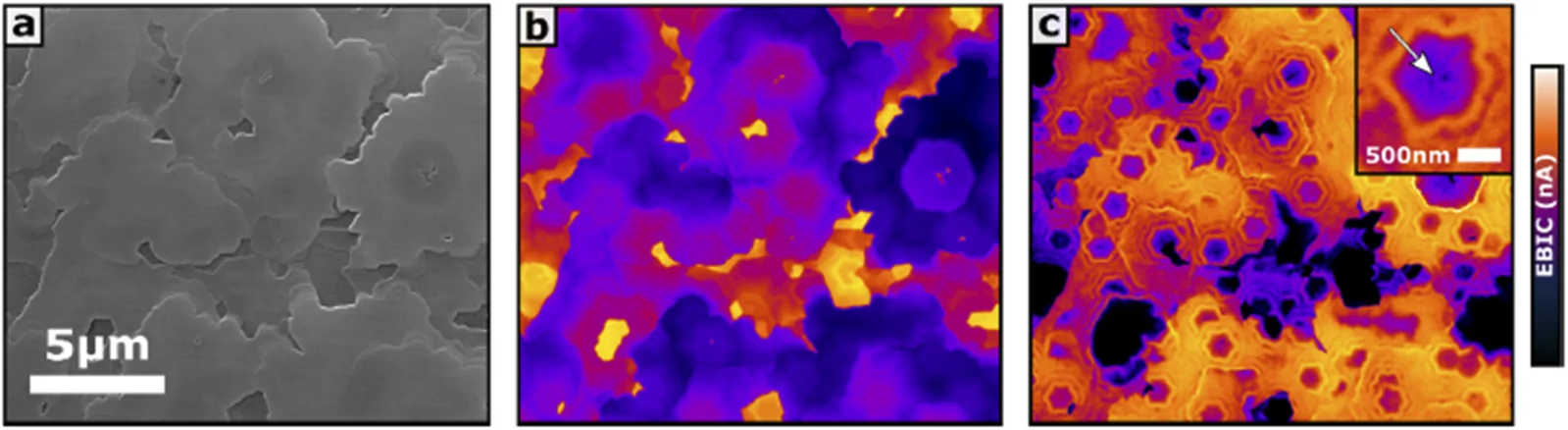
EBIC images of the same region of an AlGaN UV-emitting LED acquired at different electron beam energies. (a) Secondary electron image of the area. EBIC images at (b) 5 keV and (c) 20 keV, including an inset highlighting an extended defect. Reproduced from Cameron *et al.*,^32^ licensed under CC BY.

To exemplify this approach, we consider LEDs emitting near 260 nm and composed of quantum wells of Al_0.47_Ga_0.53_N/Al_0.60_Ga_0.40_N deposited by MOVPE on sapphire substrates.^32^ EBIC measurements were performed in both plan-view and cross-sectional geometries using a nanoprobing system within the SEM to contact n- and p-regions of the LED. For planar EBIC the size of the signals is very dependent on the overlap of the electron generation volume with the buried space charge region. Fig. 5 shows EBIC contrast arising from inhomogeneities in the ≈40 nm thick p-GaN capping layer, which can be inverted by increasing the acceleration voltage from 5 kV to 20 kV corresponding to excitation predominately above or below the junction. High spatial resolution is maintained at the higher acceleration voltages as the EBIC highlights the junction region and a network of small hexagonal and dodecagonal rings is evident. The depth resolved EBIC indicates that the hexagonal ring structure originates from a 3D growth mode around crystallographically aligned hillocks in the underlying n-contact layer. Dark spots at the centre of the rings (see inset to Fig. 5(c)) identify threading dislocations. CL images collected simultaneously reveal a related set of dark spots, with the coincidence of low signals in both EBIC and CL identifying an increase in non-radiative recombination at the extended defects. ECCI data from similar n-AlGaN layers (see below) indicates that they are screw type threading dislocations.^33^ The cross-sectional EBIC (see SI in ref. 32) highlighted a second weaker junction at an AlN/AlGaN heterojunction near the substrate, due to an additional junction region caused by bandgap discontinuity and polarization fields.

A separate report on CL-EBIC of similar UV-emitting AlGaN structures illuminated the growth mechanisms forming the active layers and provided details of the point defect populations that resulted.^34^ In this case understanding the complex star-shaped contrast in the EBIC and CL was aided by complementary AFM images of the spiral growth morphology of the AlGaN.

**Fig. 6:**
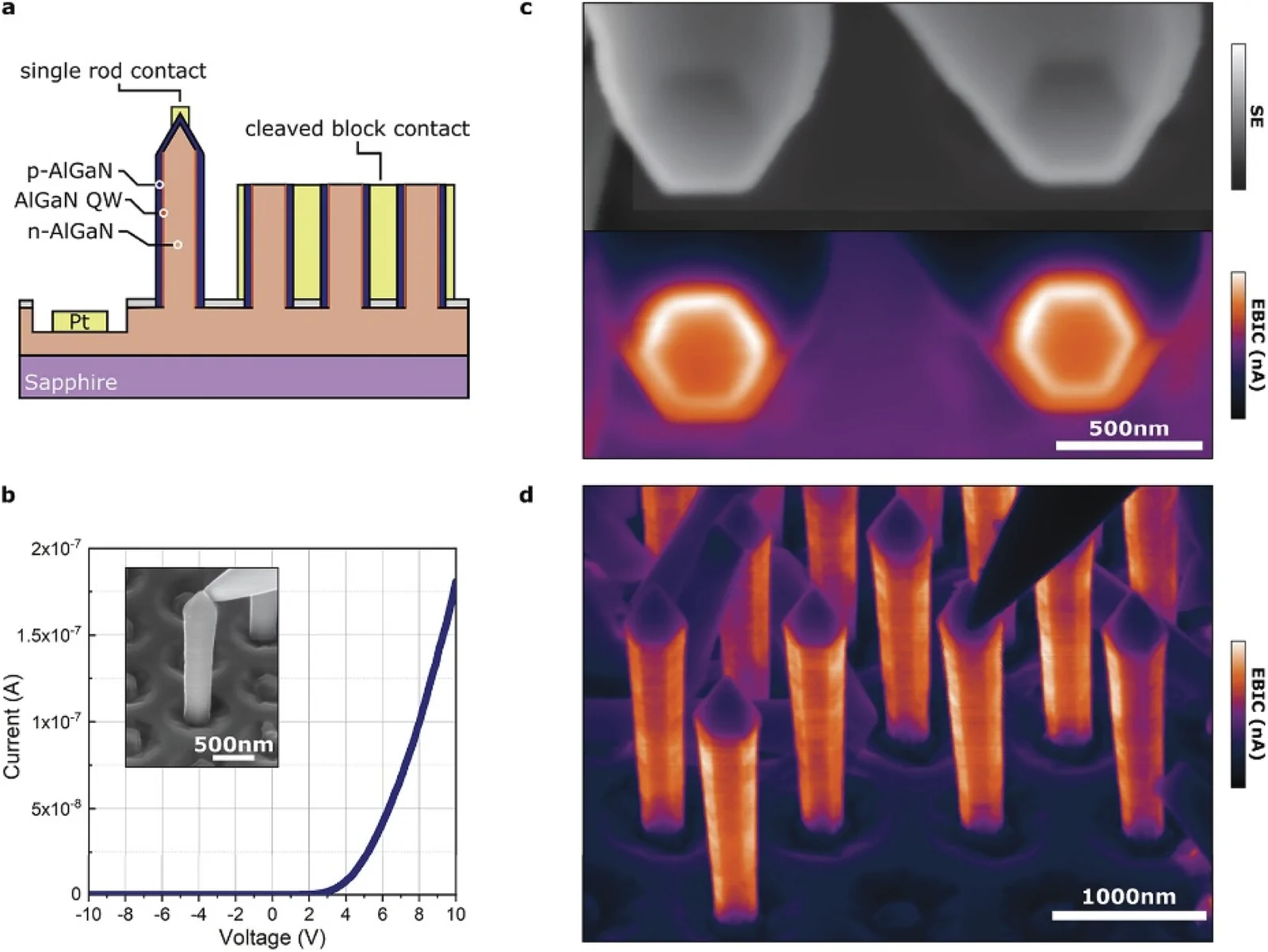
EBIC characterisation of UV-emitting core–shell LED structures. (a) Schematic of the nanorod electrical testing architecture. (b) Example *I*–*V* curve for a single rod contacted with a nanoprobe as seen in the inset. (c) SE and EBIC images of the cleaved block contact viewed from above, showing the presence of the junction around the entire circumference of the rods. (d) EBIC image of a single-rod contact, with the nanoprobe contacting from above. Reproduced from Cameron *et al.*,^35^ licensed under CC BY.

The EBIC described so far was measured from planar structures with laterally separated contacts. The circuit could be made with wire bonds to the contacts or using the nanoprobing system within the SEM chamber. Nanoprobes also facilitate the study of EBIC from more complex geometries such as nanorod LEDs.^35^ A number of the barriers to efficient UV-emitters can be reduced or overcome by these 3D-nanostructures but the geometry brings some additional measurement challenges. The SEM-based techniques described here have the resolution and sufficient flexibility in sample orientation to allow the study of individual nanorods within large arrays. For the EBIC, contact pads deposited on or around the nanorods can be contacted using a nanoprobing set-up, which allows study of the electrical properties of the nanorod LEDs as well as analysis of the electroluminescence using the CL collection system. The schematic in Fig. 6(a) shows platinum contacts created on or around core–shell UV-emitting LEDs fabricated using displacement Talbot lithography and MOVPE.^35^ The central n-doped region of the rods is contacted by a common side contact formed using a focussed ion beam (FIB). Fig. 6(b) shows electrical data measured from a single nanorod with the inset showing one of the two nanoprobes touching a p-contact deposited on the apex of the rod. This shows strong rectification with a low turn-on voltage, indicating successful formation of a p–n junction. Fig. 6(c) and (d) show two different formats of EBIC from these rods, both with one nanoprobe on the common n-contact. The first shows data from rods that were cleaved open using the FIB with a nanoprobe contacting a block p-contact. In this case the EBIC highlights the in-built electric field in a p–n junction created uniformly around the entire circumference of the hexagonal rods. The second shows an EBIC image created using a single-rod p-contact, with the nanoprobe contacting from above, and now demonstrates the uniformity of the p–n junctions down the full height of the rods and across an array of rods. The optical properties of the nanorod LEDs were investigated using CL and time-resolved CL, showing luminescence all the way down the polar sidewalls although with spots of increased intensity that could be related to features observed in TEM cross-sections of the nanorods.^35^

### Dislocation identification in AlGaN using ECCI and CL

**Fig. 7:**
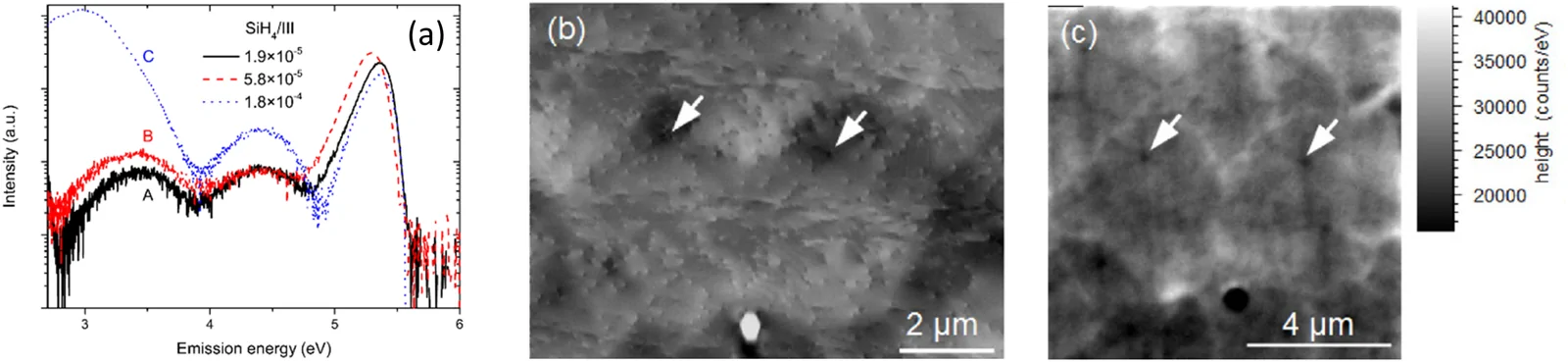
(a) CL spectra of Al_0.82_Ga_0.18_N epilayers with different levels of Si doping. (b) ECCI image and (c) image of the CL intensity of the near band edge peak at 5.3 eV for the intermediate doped sample B. Adapted from Kusch *et al.*,^33^ licensed under CC BY.

An earlier section described how ECCI can serve as a nondestructive technique for the quick analysis of the dislocation density and also give qualitative information on tilt and twist boundaries. For the ultra-wide bandgap AlGaN the ECCI, in combination with CL and X-ray microanalysis, proved to be useful for providing complementary information on composition, doping and defects. As an example, consider work on a series of Si-doped Al_0.82_Ga_0.18_N epilayers grown by MOVPE on defect-reduced AlN-on-sapphire templates.^33^ WDX measurements gave the composition and the CL spectra (Fig. 7(a)) showed a number of peaks, including near band edge peaks centred around 5.3–5.4 eV and a number of peaks related to Si-defects. The dominant defect luminescence depends on the Si-doping concentration. For lower doped samples there are clear peaks centred near 4.4 and 3.4 eV, while an additional, stronger peak appears at 3 eV for the highest doped sample. The first two peaks can be attributed to recombination of a shallow Si donor level and acceptor complexes involving oxygen antisites or interstitials.^33^ Multimode imaging using CL, secondary electrons, ECCI and AFM showed how the luminescence intensity of these peaks was not homogeneously distributed but showed a strong dependence on the topography and on the distribution of screw dislocations. The layers are composed of hillocked domains and the ECCI identified a threading dislocation with a screw component at the apex of each hillock, as shown in Fig. 7(b).

It can be concluded that the hillocks form due to spiral growth around the screw-type dislocations, which act as non-radiative centres reducing the near band edge CL intensity (Fig. 7(c)). Conversely CL maps of the defect luminescence showed the 3.4 eV to increase in intensity at the hillock apices, leading to the conclusion of increased oxygen incorporation around the dislocations.^33^

### Simultaneous mapping of CL and EBSD

EBSD generally requires the sample to be tilted at a high angle (≈70°) to the electron beam, making it difficult to measure other signals at the same time. For example, CL and EBSD could usefully complement each other, separately providing details of optical properties and strain, grain structure, *etc.*, but even if their detectors could both be positioned to see the same tilted sample surface their need to view a large solid angle means that they are likely to obscure each other. If simultaneous measurements were possible it would avoid the need for sequential scans, with the possibility of the first inducing some impact on the sample, and challenges with measuring exactly the same area. A way of achieving simultaneous EBSD and CL was demonstrated by Edwards *et al.*^36^ who presented results from UV-emitting micro-LED samples with a transparent substrate. The CL could be measured through the back of the sample at the same time as recording EBSD from the front, leading to detailed maps of the full strain tensor and CL spectra. Correlation plots then allowed subtle relationships to be drawn out, where the strain in the etched micro-LED impacted its optical emission.^36^

## Characterising 2D semiconductors using CL, ECCI and EBSD

The resolution of the SEM-based measurements is determined by the spread of the energy deposition from the incident electrons through the material as well as by diffusion of the generated electrons and holes. This can be reduced by measuring at low electron beam energy (*e.g.* 1.5 keV in ref. 4) or by cooling to reduce the diffusion coefficient.^4^ In cases where the layers contain luminescent nanostructures particularly small structures can be resolved.^37^ Measuring thin layers, such as 2D-materials, poses an additional challenge as the exciting electrons tend to pass through. However, innovations have enabled CL, ECCI and EBSD to be measured from 2D materials. Encapsulation of monolayers of transition metal dichalcogenides (TMDCs) in thicker hexagonal boron nitride (hBN) has been shown to increase the interaction volume with the electron beam and significantly increase the CL intensity from the TMCD.^38–40^ Francaviglia *et al.*^38^ investigated exciton diffusion within the hBN with a view to optimising the CL of the buried TMDC monolayers and postulated that using hBN as an indirect excitation medium could be generalised to other nanoscale semiconductors, including delicate, beam-sensitive structures. Bonnet *et al.*^39^ mapped the CL from monolayers of WS_2_ sandwiched between hBN layers using a scanning transmission electron microscope at reduced temperature (150 K) with electron energy loss spectroscopy (EELS) complementing the CL. The results demonstrated nanometre-scale localized light emission in relatively structurally homogeneous WS_2_ monolayers, which were related to local dielectric variations and charge accumulation.^39^ A very recent paper reported the use electrostatic gating to modify the CL from a WS_2_ layer encapsulated in hBN and with a graphene back gate.^40^ The spatially resolved, low temperature CL data demonstrated the ability to form locally-confined excitons within the 2D layers with potential for engineering quantum devices.

Controlling the angle of twist between different TMCD 2D layers has been shown to open the way to novel phenomena and applications. The ECCI technique discussed above for the analysis of grain structures and dislocations in bulk crystalline materials is well-suited to the study of the twist domains in these TMDC heterostructures. This was realised by Tillotson *et al.*^41^ who demonstrated ECCI of twisted domains in stacked layers of either MoS_2_ or WS_2_ using a range of different SEM detectors and showed that prior knowledge of sample crystallinity was not a prerequisite. They also showed that the twist domains could still be resolved by ECCI for both types of stacked TMDCs after encapsulation by hBN, relevant in this case as practical device architectures need capping layers or top-gates.

The EBSD technique in the SEM has also been successfully applied to TMDC materials. Zhang *et al.*^42^ used a direct electron detector to collect EBSD data from monolayer, bilayer and trilayer WSe_2_ films in the standard geometry and also in reflection Kikuchi diffraction (RKD) geometry with the detector mounted below the pole-piece. The minimum number of layers required to give sufficient scattering events for a usable Kikuchi diffraction pattern was investigated, with diffraction patterns clearly visible even for only two layers of WSe_2_.^42^

## Conclusions

The wide range of material characterisation techniques possible using a scanning electron microscope has been presented and explored with a view to providing information on the optical, structural, electrical properties of semiconductor materials. Example results from visible InGaN light-emitters, perovskite solar absorber layers, UV-emitting aluminium gallium nitride and 2D materials have been presented to highlight the information that can be generated by combining techniques, often with simultaneous measurement. Cathodoluminescence (CL) hyperspectral imaging is used to assess the optical properties and wavelength dispersive X-ray (WDX) provides compositional information. Electron channeling contrast imaging (ECCI) and electron backscatter diffraction (EBSD) furnish structural information, and electron beam induced current (EBIC) gives electrical information. Details of the defect populations, grain structure, and strains within a range of emerging semiconductor materials are discussed to highlight the opportunities for characterisation to provide information on application-relevant properties.

The power of correlative or multi-modal SEM is being harnessed more and more by a range of groups. Going forwards there are exciting opportunities arising from the increasing availability of direct electron detectors, allowing new measurement geometries and higher measurement sensitivity. Improved detectors and cryogenic cooling not only enhance the signal but can also reduce measurement times, especially important for detailed mapping, which increases the reliability of data from beam sensitive materials. These developments can also reduce the electron beam spread within the material and facilitate high resolution mapping, with the reduced measurement times extending this to larger areas. Theoretical advances, such as compressive sensing, could also positively impact the data collection and then there is the rapidly growing power of machine learning and other AI techniques, both for extracting more information from the data as well as in guiding or transforming the measurement strategy.

## Conflicts of interest

There are no conflicts to declare.

## Data Availability

The data contained in this article is available through the papers cited after each figure or in the text.
